# Educating Pharmacists on the Risks of Strong Opioids With Descriptive and Simulated Experience Risk Formats: A Randomized Controlled Trial

**DOI:** 10.1177/23814683211042832

**Published:** 2021-09-27

**Authors:** Odette Wegwarth, Stefan Wind, Eva Goebel, Claudia Spies, Joerg J. Meerpohl, Christine Schmucker, Erika Schulte, Edmund Neugebauer, Ralph Hertwig

**Affiliations:** Max Planck Institute for Human Development, Center for Adaptive Rationality, Berlin, Germany; Charité—Universitätsmedizin Berlin, Heisenberg Professorship for Medical Risk Literacy and Evidence-Based Decisions; Berlin Chamber of Pharmacists, Berlin, Germany; Berlin Chamber of Pharmacists, Berlin, Germany; Charité—Universitätsmedizin Berlin, Department of Anesthesiology and Operative Intensive Care Medicine, Berlin, Germany; University of Freiburg, Faculty of Medicine & Medical Center, Institute for Evidence in Medicine (for Cochrane Germany Foundation), Freiburg, Germany; Cochrane Germany, Cochrane Germany Foundation, Freiburg, Germany; University of Freiburg, Faculty of Medicine & Medical Center, Institute for Evidence in Medicine (for Cochrane Germany Foundation), Freiburg, Germany; Charité—Universitätsmedizin Berlin, Department of Anesthesiology and Operative Intensive Care Medicine, Berlin, Germany; Brandenburg Medical School Theodore Fontane, Neuruppin, Germany; Max Planck Institute for Human Development, Center for Adaptive Rationality, Berlin, Germany

**Keywords:** strong opioids, pharmacists’ risk perception, pharmacists’ opioid counseling, drug safety, description-experience gap

## Abstract

**Objectives.** High opioid prescription rates in the United States and Europe suggest miscalibrated risk perceptions among those who prescribe, dispense, and take opioids. Findings from cognitive decision science suggest that risk perceptions and behaviors can differ depending on whether people learn about risks by experience or description. This study investigated effects of a descriptive versus an experience-based risk education format on pharmacists’ risk perceptions and counseling behavior in the long-term administration of strong opioids to patients with chronic noncancer pain. **Methods.** In an exploratory, randomized controlled online trial, 300 German pharmacists were randomly assigned to either a descriptive format (fact box) or a simulated experience format (interactive simulation). **Primary Outcome Measures.** 1) Objective risk perception, 2) subjective risk perception, and 3) intended and 4) actual counseling behavior. **Results.** Both risk formats significantly improved pharmacists’ objective risk perception, but pharmacists exposed to the fact box estimated the benefit-harm ratio more accurately than those exposed to the simulation. Both formats proved equally effective in adjusting pharmacists’ subjective risk perception toward a better recognition of opioids’ harms; however, pharmacists receiving the simulation showed a greater change in their actual counseling behavior and higher consistency between their intended and actual counseling than pharmacists receiving the fact box. **Conclusion.** The simulated experience format was less effective than the descriptive format in improving pharmacists’ objective risk perception, equally effective in motivating pharmacists to counsel patients on less risky treatment alternatives and more effective in changing the reported actual counseling behavior. **Implications.** These exploratory findings provide important insights into the relevance of the description-experience gap for drug safety and raise questions for future research regarding the specific mechanisms at work.

## Introduction

Prescribing opioids can make good sense. Most patients experience adequate pain reduction when strong opioids are used to treat acute or cancer pain.^1^ However, there is little and insufficient evidence that strong opioids—defined as step III opioids on the World Health Organization pain ladder—are effective in the long term or superior to other analgesics in patients with chronic noncancer pain.^2^ Despite this lack of supporting evidence,^3,4^ strong opioids are commonly prescribed to this patient group, with increasing prescription rates in Europe (e.g., the Netherlands,^5^ Germany,^6–8^ the United Kingdom^9^) and a full-blown opioid epidemic in the United States.^10,11^ In Germany alone, about 80% of patients receiving strong opioids long term (>3 months) have chronic noncancer pain,^6^ even though a national evidence- and consensus-based clinical practice guideline (S3)^12^ cautions against the long-term use of strong opioids in this group and recommends that they be used only after thorough assessment of the benefits and harms and with close monitoring.^12^

One reason for this non-evidence-based use of strong opioids might be that many health care professionals and patients have difficulties understanding medical statistics,^13–23^ resulting in unrealistic views of the benefit-harm ratios of medical interventions.^24–26^ Although transparent statistical formats (e.g., absolute instead of relative risks)^27^ and visualizations (e.g., fact boxes)^28,29^ have been developed to improve the communication of medical statistics,^23,30^ findings suggest that not everybody benefits from these educative formats.^24^ An explanation for this somewhat unexpected finding may come from research in cognitive decision sciences, which has shown that risk perceptions and behaviors can be shaped by two learning paths: through personal experience (e.g., taking a medication and experiencing its consequences firsthand) and through descriptive information (e.g., medical evidence and statistics, guidelines, patient information). Depending on whether an individual has experienced a risk and/or received a description of it, they may behave as if they overestimate, underestimate, or correctly estimate the risk. For instance, an individual who has personally experienced a rare but life-threatening risk may subsequently act as if that risk were significantly higher than is objectively the case.^31,32^ Conversely, an individual who experiences many episodes of a risky behavior without the risk materializing—because “experience samples” are often too small to permit the observation of a rare and possibly cumulative risk (e.g., in substance use)—may behave as if they underestimate or underweight the risk.^33–35^ If the experience of risk impacts risk perceptions and behaviors,^36^ could simulated experience be harnessed to educate and inform people as witnessed in areas such as financial decision making or probabilistic reasoning?^37–39^

To examine the effects of the two modes of learning about risks in the field of drug safety, we set up four randomized controlled trials (RCTs) under the umbrella of the ERONA project. The RCTs investigated four groups involved in the long-term administration of strong opioids: family physicians, physicians specialized in pain therapy, patients with chronic (≥3 months) noncancer pain, and pharmacists who regularly dispense narcotic substances. Here, we report results from the ERONA trial with pharmacists on the effects of an educative intervention involving either a simulated experience format (interactive simulation) or a descriptive format (fact box) on their 1) objective risk perception, 2) subjective risk perception, 3) intended counseling behavior, and 4) actual counseling behavior at 9-month follow-up.

## Methods and Analysis

The ERONA project is funded by a grant from the German Federal Ministry for Health under the guideline “Risk perception and risk behavior among stakeholders involved in settings of drug safety concern.” We described the designs and methods in detail in a study protocol^35^ that has not since been amended, registered the trial at the German Clinical Trials Register (DRKS00020358), made trial information public on the Open Science Framework (OSF), and adhered to the CONSORT checklist. In brief, the study is based on an exploratory independent RCT with two parallel online intervention arms. Data were collected before intervention at baseline (T0), immediately after intervention (T1), and 9 months after intervention (T2). The Institutional Ethics Board of the Max Planck Institute for Human Development, Berlin (Germany), approved the study (Ethic Approval ID: A 2020-05).

### Sample Frame and Sample Size

The sample frame comprised accredited offline and online panels of IPSOS Health (Nuremberg, Germany) consisting of general populations of pharmacists. To detect a 15% difference in a two-tailed test with a 5% level of significance and a power of 80%, the trial required 150 participants per intervention arm (for details, see Wegwarth et al.^40^). IPSOS started enrolment for the first wave (T1) in April 2020 and concluded it in August 2020. The enrolment for the 9-month follow-up (T2) began in January 2021 and was completed in April 2021. Eligibility was determined by a set of screening questions. Randomization was achieved by simple randomization. Participants were blind to the type of intervention they received.

IPSOS approached 2679 eligible pharmacists, of whom a total of 369 started the trial upon invitation; 69 abandoned it prematurely before randomization to either intervention, leaving 300 participants (150 per intervention arm; CONSORT flow chart, Supplementary Material). Nine months after participation in the first wave, participants were approached again and asked solely about their actual counseling behavior. IPSOS approached only those (*n* = 184) who had stated at baseline (T0) that they actively counsel chronic noncancer pain patients with a long-term prescription of strong opioids on treatment alternatives, because the primary outcome of “counseling behavior” could be retrieved at T0, T1, and T2 for those pharmacists only. Of these 184 pharmacists (fact box: *n* = 91, simulation: *n* = 93), 133 (fact box: *n* = 72, simulation: *n* = 61) participated in the follow-up. Informed consent was acquired prior to the study. Participation was monetarily reimbursed.

### Interventions

**Figure 1:**
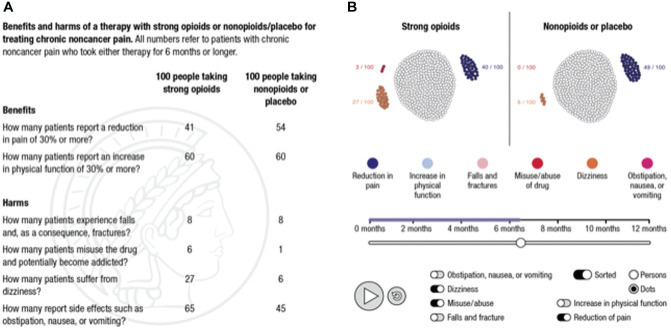
In the descriptive risk education format, the numerical values were concealed and participants had to move the mouse pointer over the respective cells of the fact box to access the information. In the interactive simulation, participants could observe changes over time by pressing the play button or by moving the horizontal slider to look at particular moments in time, they could explore specific risks by activating and deactivating the respective buttons, and they could sort the presentation of information.

A fact box format was used for the descriptive intervention and an interactive simulation was used for the simulated experience intervention (see Figure 1).

Both risk education interventions presented information on the benefit-harm ratio of the long-term administration of strong opioids in patients with chronic noncancer pain as absolute risks, adjusted to the same denominator (here: per 100 people), and compared with a control group (here: nonopioids or placebo). Numerical estimates of the benefits and harms were based on a systematic rapid review^41^ conducted for the purposes of this RCT by the Institute for Evidence in Medicine (for the Cochrane Germany Foundation).^42^

The two risk education formats differed in several respects: The interactive simulation presented information on the benefits and harms of strong opioids and of nonopioids/placebo interactively and sequentially, which allowed participants to directly observe change in the outcomes over time and to explore each outcome separately by using interactive filter functions. Fact boxes typically present information on benefits and harms of each treatment in tabular, static form. To address the different levels of interactivity of the two educative formats in this RCT, we implemented the fact box using MouseLab^43^ (www.mouselabweb.org): Participants had to move the mouse pointer over cells of the fact box to access the numerical information about each benefit and harm.

### Survey Questionnaire

Before completing the survey questionnaires, participants provided demographic information (age, gender, years in practice, region of practice).

The primary endpoints surveyed at baseline (T0) and immediately after intervention (T1) were 1) objective risk perception, 2) subjective risk perception, and 3) reported baseline counseling on alternatives to strong opioids (T0) and reported intended counseling on alternatives to strong opioids (T1). The primary endpoint investigated at 9-month follow-up (T2) was 4) reported actual counseling on alternatives to strong opioids. 1) Objective risk perception was operationalized by a series of six questions requiring participants to provide a specific numerical estimate for each of the outcomes (benefits/harms) presented in the intervention (see Figure 1). 2) Subjective risk perception was measured using a 5-point Likert-type scale with five options reaching from “The benefits of strong opioids clearly outweigh the harms” to “The harms of strong opioids clearly outweigh the benefits.” 3) It is neither standard nor mandatory for pharmacists to actively counsel chronic noncancer pain patients with a long-term prescription of opioids on alternative therapies. Pharmacists were therefore asked at baseline (T0) whether they actively counsel chronic pain patients on long-term opioid prescription. If their answer was positive, they were presented with four treatment alternatives—physiotherapy, lifestyle changes (e.g., reactivating social life), psychotherapy, and multimodal pain therapy—and asked to indicate the number of patients out of 100 to whom they currently recommend each treatment alternative (“counseling at baseline”) by moving a slider between 0 and 100. After intervention (T1), only the pharmacists who reported at baseline that they actively counseled patients were presented with the four treatment alternatives again and asked to indicate any increase or decrease in the number of patients out of 100 to whom they intended to recommend each treatment alternative in the future (“intended counseling”) by readjusting the slider. 4) To investigate pharmacists’ actual counseling behavior at the 9-month follow-up, we presented them with their responses on treatment alternatives at T1 and asked them to indicate any increase or decrease in their actual relative to intended counseling behavior by moving the slider. As moderator variable, we assessed participants’ medical risk literacy by administering an adapted version of the validated Critical Risk Interpretation Test (CRIT).^44^ To prevent participants from abandoning the interventions prematurely and based on time estimates retrieved from pilot testing, the “move on” button was deactivated for 3 minutes for both interventions.

The phrasing of the questions was piloted with 12 German pharmacists to ensure readability and relevance, and revised on the basis of their feedback.

### Effect Measures

To analyze 1) objective risk perception, we compared mean numerical estimates of each benefit and harm at T0 and T1 and calculated mean differences between the two intervention conditions. To analyze 2) subjective risk perception, we investigated change in the Likert-scale judgments from T0 to T1. To evaluate the influence of the interventions on pharmacists’ counseling behavior, we calculated means and mean differences between baseline counseling behavior (T0) and counseling behavior at 9-month follow-up (T2) and tested for the implementation of intended behavior^45^ by investigating for the consistency between physicians’ reported intended prescription behavior at T1 and their actual counseling behavior at T2.

### Data Analysis Plan

The online questionnaire did not permit item nonresponse; there were thus no missing variables. Differences between the intervention groups were assessed using independent sample *t* tests or Mann-Whitney *U* tests (for continuous variables) or χ^2^ tests (for categorical variables). Differences within each group (before/after comparisons) were assessed using dependent sample *t* tests (for continuous variables) or Wilcoxon and McNemar’s tests (for ordinal data). Independent predictors (e.g., medical risk literacy) of risk perception and counseling behavior were analyzed using regression analysis. Data were stored and analyzed with IBM SPSS Statistics 26. To control for nonresponse bias,^46^ we compared the demographic characteristics of respondents and nonrespondents.^47,48^

## Results

### Sample Characteristics

Table 1 reports the distribution of age, gender, years in practice, and region of practice for all pharmacists who finished the survey (respondents) and for those who abandoned the survey prematurely (nonrespondents). Relative to respondents, nonrespondents tended to be younger and less experienced in terms of years in practice. Slightly more of them were female and more of them came from the south of Germany. Respondents exposed to the fact box format and respondents exposed to the simulated experience formats did not differ in distribution of age, gender, years in practice, and region of practice.

**Table 1:** Demographic Characteristics of Respondents (Survey Sample) and Nonrespondents (Pharmacists Who Abandoned the Survey Prematurely)

	Respondents, %^a^	Nonrespondents, %^a^
Female	41	44
Age (in years)		
<20	0	0
20–39	20	28
40–59	58	57
60–79	22	14
≥80	0	1
Years in practice		
<10	14	22
10–19	33	28
20–29	35	33
30–39	18	17
≥40	0	0
Region of practice		
North Germany	26	22
East Germany	23	22
South Germany	26	30
West Germany	26	26

a Percentages are rounded and may not total 100.

### Objective Risk Perception

**Figure 2:**
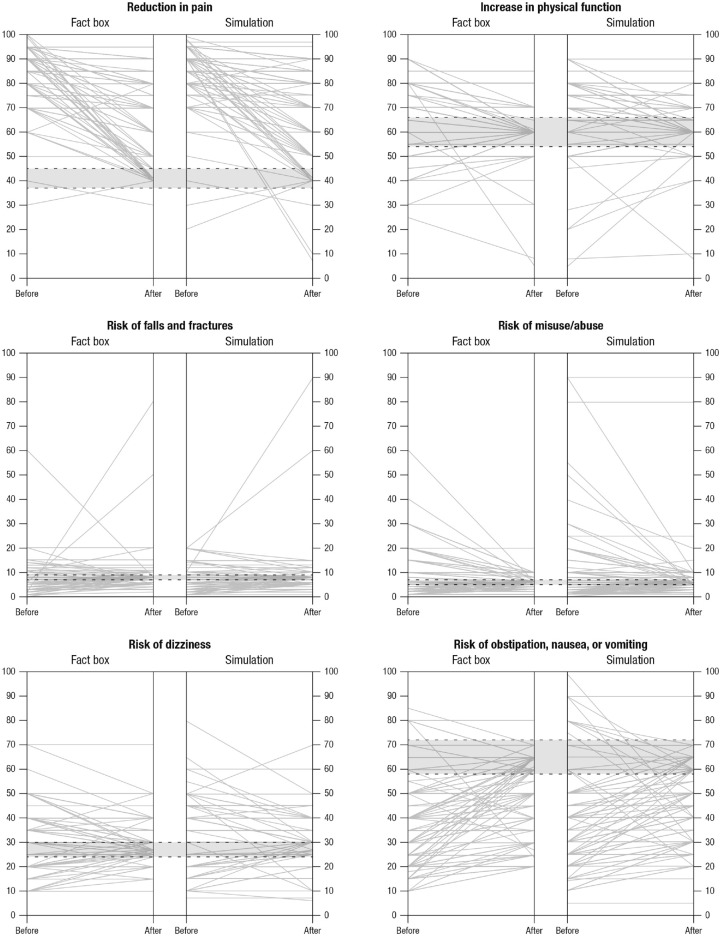
Pharmacists’ risk estimates for each benefit and harm outcome at baseline (T0) and after intervention (T1) by group (descriptive format [fact box] and simulated experience-based format [interactive simulation]). The gray area within the dashed lines shows correct estimates falling within the ±10% margin of error.

Both the simulated experience and the descriptive format significantly improved pharmacists’ objective risk perception of the benefits and harms of long-term administration of strong opioids (see Table 2). Participants in the descriptive condition, however, arrived more often at accurate numerical estimates than did participants in the simulated experience condition (see Table 2). Comparison of mean estimates across the two conditions found statistically significant differences for the estimates of “reduction in pain” (*t*[295.6] = −2.93, *P* < 0.01) and “risk of obstipation, nausea, and vomiting” (*t*[295.6] = −2.07, *P* = 0.05) in favor of the fact box condition (see Table 2). Figure 2 illustrates pharmacists’ risk estimates for each outcome before and after intervention by group; correct estimates falling within the ±10% margin of error are shown in the gray area.

**Table 2:** Influence of the Descriptive Versus the Experience-Based Risk Education Format on Pharmacists’ Objective Risk Perception About the Benefits and Harms of Long-Term Administration of Strong Opioids (Baseline [T0] v. Immediately After Intervention [T1])

	Risk Formats										
	Fact Box (Descriptive Format) (*n* = 150)			Correct Estimate	Interactive Simulation (Simulated Experience Format) (*n* = 150)			Fact Box v. Simulation			
	Before Intervention (T0): Mean estimate (SD)	After intervention (T1): Mean estimate (SD)	P* (Effect Size *r*)		Before Intervention (T0): Mean estimate (SD)	After Intervention (T1): Mean estimate (SD)	P* (Effect Size *r*)	Before Intervention		After (T1) Intervention	
								Mean Difference	*P* *	Mean Difference	P* (Effect Size *r*)
Reduction in pain	84.3 (10.8)	66.8 (19.3)	<0.01* (0.66)	41	84.5 (11.3)	73.2 (18.0)	<0.01* (0.55)	0.2	0.90	6.8	<0.01* (0.17)
Increase in physical function	65.0 (14.3)	59.6 (10.2)	<0.01* (0.40)	60	65.6 (14.8)	61.9 (11.0)	<0.01* (0.32)	0.6	0.75	2.3	0.06 (0.11)
Risk of falls/fractures	6.9 (5.7)	8.4 (7.3)	0.03* (0.17)	8	6.9 (4.0)	8.1 (8.4)	0.05* (0.16)	0.0	0.96	0.3	0.76 (0.02)
Risk of misuse/addiction	6.7 (8.3)	5.5 (2.8)	0.05* (0.16)	6	8.5 (14.1)	6.5 (9.8)	0.01* (0.20)	1.8	0.19	1.0	0.22 (0.07)
Risk of dizziness	33.0 (11.5)	31.3 (8.0)	0.02* (0.20)	27	33.2 (11.9)	32.5 (9.4)	0.30 (0.10)	0.8	0.89	1.2	0.24 (0.07)
Risk of nausea, obstipation, vomiting	37.5 (17.7)	48.7 (15.9)	<0.01* (0.58)	65	39.5 (18.6)	45.1 (14.5)	<0.01* (0.35)	3.0	0.34	3.6	0.05* (0.13)

* Significance level is two-tailed and set at 0.05.

### Subjective Risk Perception

**Figure 3:**
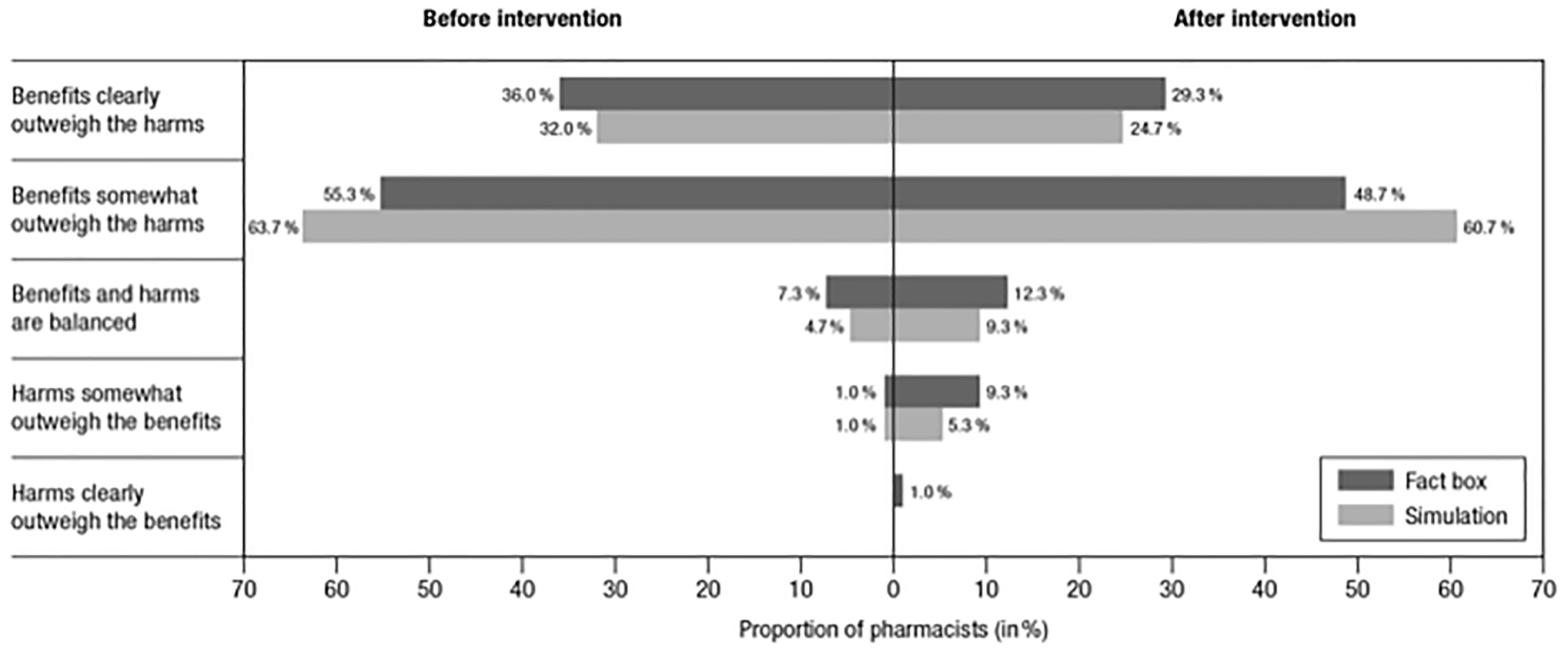
Subjective risk perception before and after interventions.

Both risk education formats also proved effective in changing pharmacists’ subjective risk perception of the benefit-harm ratio of the long-term administration of strong opioids in patients with chronic noncancer pain (see Figure 3). The proportion of pharmacists who believed that the harms are on par with or outweigh the benefits significantly increased in both the fact box condition (absolute increase: 14.3%; *z* = −4.63, *P* < 0.01) and the interactive simulation condition (absolute increase: 10.0%; *z* = −4.45, *P* < 0.01). Subjective risk perception did not differ between the two intervention groups (*P* = 0.76).

### Intended and Actual Counseling Behavior

After intervention (T1), the proportion of pharmacists who said they intended to proactively counsel patients with chronic noncancer pain on treatment alternatives did not differ between intervention groups (*P* = 0.97): 60.7% (*n* = 91) in the fact box and 62% (*n* = 93) in the interactive simulation condition. Responses to the question tapping the proportion of patients to whom pharmacists intended to recommend each of four treatment alternatives indicated that lifestyle changes ranked highest in both the fact box condition and the simulation condition (mean [M]: 69.4% [SD: 21.3] v. 68.9% [SD: 20.7], *P* = 0.87), followed by physiotherapy (M: 26.8% [SD: 25.0] v. 30.9% [SD: 27.2], *P* = 0.29), psychotherapy (M: 14.6% [SD: 20.7] v. 14.3% [SD: 19.4], *P* = 0.91), and multimodal therapy (M: 7.3% [SD: 13.2] v. 7.1% [SD: 13.4], *P* = 0.94).

This hierarchy in recommendation of treatment alternatives remained consistent at the 9-month follow-up. The two intervention groups did not differ from each other in the reported actual counseling behavior for any of the four treatment alternatives. However, there were differences within the intervention groups in terms of the actual change between the counseling behavior reported at baseline (T0) and at the 9-month follow-up (T2) and in terms of consistency between intended (T1) and actual counseling behavior (T2). While both interventions led to some notable differences in the mean counseling rates of less risky therapy alternatives between T0 and T2, the simulation intervention was more effective (Table 3). Pharmacists presented with the simulated experience condition showed an increased mean counseling rate at T2 for three out of the four alternative therapy options: physiotherapy (*t*[60] = −2.83, *P* = 0.006), lifestyle changes (*t*[60] = −3.03, *P* = 0.004), and psychotherapy (*t*[60] = −2.48, *P* = 0.016). The counseling behavior of pharmacists presented with the fact box changed significantly only for two alternative options from baseline (T0) to the 9-month follow-up (T2), with one option presenting a decreased mean counseling rate: lifestyle changes (*t*[71] = −3.13, *P* = 0.003) and psychotherapy (*t*[71] = 2.35, *P* = 0.021). Note that, depending on the alternative therapy, between 69.4% and 79.2% of pharmacists in the fact box condition and between 67.2% and 90.2% of pharmacists in the simulated experience condition did not report a change in their counseling behavior between T0 and T2, which resulted in overall differences between the means that appear small despite sometimes being significant. Comparing the mean differences from T0 to T2 for only those pharmacists who reported a change in their counseling behavior led to more notable differences—for the fact box: physiotherapy: mean difference (MD) −1.85, SD: 14.79; lifestyle changes: MD: 9.33, SD: 8.21; psychotherapy: MD: −5.60, SD: 10.28; multimodal therapy: MD: −4.44, SD: 9.40; for the simulation: physiotherapy: MD 5.50, SD: 3.60; lifestyle changes: MD: 10.85, SD: 8.77; psychotherapy: MD: 6.90, SD: 11.25; multimodal therapy: MD: −1.17, SD: 2.23.

**Table 3:** Differences Between Reported Counseling Behavior at Baseline (T0) and the Reported Actual Counseling Behavior at 9-Month Follow-up (T2) for Pharmacists Who Participated in Both Waves^a^

Recommended Treatment Alternative	Fact Box Condition (*n* = 72)			Simulated Experience Condition (*n* = 61)		
	Reported at Counseling at Baseline (T0), Mean (SD)	Reported Actual Counseling (T2), Mean (SD)	P* (Effect Size *r*)	Reported Counseling at Baseline (T0), Mean (SD)	Reported Actual Counseling (T2), Mean (SD)	P* (Effect Size *r*)
Physiotherapy	25.0 (22.6)	24.1 (22.6)	0.573 (0.05)	25.9 (23.9)	26.8 (23.8)	0.006*↑ (0.34)
Lifestyle change	65.8 (21.3)	67.7 (20.7)	0.003*↑ (0.35)	64.9 (19.0)	67.2 (18.8)	0.004*↑ (0.36)
Psychotherapy	13.7 (20.7)	12.0 (19.4)	0.021*↓ (0.27)	8.84 (11.6)	11.1 (12.4)	0.016*↑ (0.30)
Multimodal therapy	6.4 (14.4)	5.4 (9.0)	0.080 (0.21)	5.6 (11.5)	5.5 (11.5)	0.226 (0.16)

a Mean (M) and standard deviation (SD) of the reported number of patients out of 100 with chronic noncancer pain being counseled on either of the respective treatment options. Arrows indicate the direction of significant change from T0 to T2.

* Significance level is two-tailed and set at 0.05.

Compared to the descriptive format, the simulated experience format also resulted in a higher propensity to implement intended behavior, measured in terms of the consistency between intended (T1) and actual counseling behavior (T2; Table 4). Within the simulated experience condition, intended and actual counseling rates differed for none of the therapy options—that is, the reported actual counseling behaviors were consistent with the intentions. Within the description condition, however, intended and actual counseling rates differed for three out of the four therapy options (Table 4), with reported actual counseling rates on alternative therapy options being lower than intended at T1.

**Table 4:** Consistency Between the Reported Intended (at T1) and the Reported Actual Counseling Behavior at 9-Month Follow-Up (T2) for Pharmacists Who Participated in Both Waves^a^

Recommended Treatment Alternative	Fact Box Condition (*n* = 72)			Simulated Experience Condition (*n* = 61)		
	Reported Intended Counseling (T1), Mean (SD)	Reported Actual Counseling (T2), Mean (SD)	P* (Effect Size *r*)	Reported Intended Counseling (T1), Mean (SD)	Reported Actual Counseling (T2), Mean (SD)	P* (Effect Size *r*)
Physiotherapy	26.8 (24.5)	24.1 (22.6)	0.013 (0.29)	27.1 (24.5)	26.8 (23.8)	0.494 (0.09)
Lifestyle change	67.3 (21.3)	67.7 (20.7)	0.540 (0.06)	66.9 (18.3)	67.2 (18.8)	0.740 (0.04)
Psychotherapy	14.6 (21.5)	12.0 (19.4)	0.004 (0.34)	10.4 (12.7)	11.1 (12.4)	0.184 (0.22)
Multimodal therapy	7.3 (14.4)	5.4 (9.0)	0.019 (0.27)	6.3 (12.6)	5.5 (11.5)	0.061 (0.24)

a Mean (M) and standard deviation (SD) of the reported number of patients out of 100 with chronic noncancer pain who receive opioids long term to whom pharmacists would recommend the respective alternative treatment.

* Significance level is two-tailed.

### Influence of Medical Risk Literacy and Demographic Variables

Overall, pharmacists displayed relatively high levels of medical risk literacy: 86.7% answered three or more of the five CRIT questions correctly. We did not find any association between the primary outcomes and medical risk literacy or demographic variables such as gender, years in practice, or region of practice (regression tables, Supplementary Material).

## Discussion

Within our exploratory RCT, we found that both risk education formats were effective in recalibrating pharmacists’ objective perceptions of opioids’ benefits and harms, by reducing over- and underestimations and boosting more correct estimation. The descriptive format, however, was better at correcting erroneous risk estimations. One potential explanation for this finding—which is not in line with findings from some other domains^33,37^—might be that pharmacists and health professionals in general are considerably more likely to be familiar with tabular presentations of risk information such as fact boxes (e.g., side effect tables in package leaflets) than with interactive simulations. Given that our RCT was conducted during the COVID-19 pandemic, a challenging time for health care professionals, it seems likely that pharmacists found it easier to attend to a familiar format than to an unknown format. The observation that only two of the 150 pharmacists in the simulation condition ran the simulation more than once and 13 made use of filter functions, which means that only 10% of the pharmacists harnessed one or the other additional information potential offered by the simulation, supports this assumption.

We also found that both interventions proved effective in improving pharmacists’ subjective perceptions toward a more realistic view of the benefit-harm ratio; here, there was no difference between the two formats. In other words, the fact that pharmacists in the fact box condition produced more correct numerical estimates on specific benefits and harms of strong opioids than did pharmacists in the simulation condition did not translate into meaningful differences in terms of subjective risk evaluation.

Likewise, it did not translate into any observable differences in intended counseling behavior between the two groups: Pharmacists in both intervention groups reported equal intentions to counsel their patients on less risky treatment alternatives. The simulated experience format did, however, outperform the descriptive format in terms of actual reported counseling behavior: While intended and actual rates of recommending for each of the four treatment alternatives did not differ in the interactive simulation group, actual rates were lower than intended rates for three of the four alternative treatments in the fact box group. The simulated experience format was also more effective in changing pharmacists’ counseling behavior toward recommending more alternative therapy options. We can only speculate about why the consistency between pharmacists’ intended and self-reported actual counseling behavior, as well as the actual change in counseling, was higher in the interactive simulation condition than in the fact box condition. In contrast to descriptive formats, interactive simulations allow participants to sequentially observe the occurrence (and potential disappearance) of a drug’s benefits and/or harms over time. Participants can thus observe, for example, that a drug can initially have potent benefits that decrease with time, while rare but serious harms may emerge over time. Insights into the sequential dynamics behind the benefit-harm ratio—insights that may also more closely mirror what pharmacists observe in their daily practice—might trigger stronger implementation intentions.^45^ As our RCT is, to our knowledge, the first investigation of the effects of different modes of learning about risks on actual behavior and intention-behavior consistency, more work is needed to replicate these findings and to better understand the underlying cognitive mechanisms.

Our study has limitations. First, our results are based on a convenience sample, which may affect generalizability of results. Our nonrespondent analysis of those who left the survey prematurely suggested some differences in age, gender, and region between respondents and nonrespondents. Second, we do not know why some pharmacists did not revise their initial estimates although they diverged from the scientific evidence presented. Time pressure due to the COVID-19 pandemic may have limited pharmacists’ capacity to fully attend to the educational material. We can, however, largely rule out the possibility that they did not know how to interpret the data presented: The information was presented in accordance with current guidelines for evidence-based health information and there is evidence that fact boxes are effective even for laypeople with low literacy levels.^29,30^ Third, while each intervention was introduced in a short tutorial on its interactive functions, which proved effective in pilot testing, we cannot exclude the possibility that some participants did not fully understand how to use and navigate through the interactive features of the interventions. Fourth, to achieve some degree of comparable interactivity between the descriptive and the simulated experience format, the descriptive format in our study was not static, as is usually the case. Instead, it was interactive: It required participants’ active involvement by moving the mouse pointer to access each of the numerical values. While the simulated experience intervention offered comparable chances for active involvement—for example, by exploring different risk information separately using interactive filter functions—the only required function for participants to access the risk information was to press the “play” button in order to start the simulation; the use of all other features was optional. Further research is required to better understand to what extent the superiority of an intervention in a given outcome is driven by required active involvement and other features.

These limitations notwithstanding, our RCT is the first exploratory trial on the description-experience gap^35^ in the field of drug safety. It provides initial and novel evidence that two promising tools—one description-based, one simulated experience-based—exist that can affect risk-related outcomes positively, but also differently: While both tools can be used to transparently educate pharmacists about a potent but high-risk drug—with the descriptive tool being potentially more effective—the simulated experience-based tool might be better suited to prompting pharmacists to recommend less risky treatment alternatives. These exploratory findings provide important insights into the relevance of the description-experience gap to drug safety. They also raise questions about what mechanisms work in what way. Our RCT provides a starting point for future researchers interested in drug safety to study the influence of different potential mechanisms in greater detail.

## Supplemental Material

sj-docx-1-mpp-10.1177_23814683211042832 – Supplemental material for Educating Pharmacists on the Risks of Strong Opioids With Descriptive and Simulated Experience Risk Formats: A Randomized Controlled Trial Click here for additional data file. Supplemental material, sj-docx-1-mpp-10.1177_23814683211042832 for Educating Pharmacists on the Risks of Strong Opioids With Descriptive and Simulated Experience Risk Formats: A Randomized Controlled Trial by Odette Wegwarth, Stefan Wind, Eva Goebel, Claudia Spies, Joerg J. Meerpohl, Christine Schmucker, Erika Schulte, Edmund Neugebauer and Ralph Hertwig in MDM Policy & Practice

## References

[1] Wiffen PJ Wee B Derry S Bell RF Moore RA. Opioids for cancer pain: an overview of Cochrane reviews. Cochrane Database Syst Rev. 2017;(7):CD012592. 10.1002/14651858.CD012592.pub2 PMC6483487 28683172

[2] Busse JW Wang L Kamaleldin M, et al. Opioids for chronic noncancer pain: a systematic review and meta-analysis. JAMA. 2018;320(23):2448–60. 10.1001/jama.2018.18472 PMC6583638 30561481

[3] Porter J Jick H. Addiction rare in patients treated with narcotics. N Engl J Med. 1980;302:123. 10.1056/nejm198001103020221 7350425

[4] Portenoy RK Foley KM. Chronic use of opioid analgesics in non-malignant pain: report of 38 cases. Pain. 1986;25(2):171–86. 10.1016/0304-3959(86)90091-6 2873550

[5] Kalkman GA Kramers C van Dongen RT van den Brink W Schellekens A. Trends in use and misuse of opioids in the Netherlands: a retrospective, multi-source database study. Lancet Public Health. 2019;4(10):e498–e505. 31444001 10.1016/S2468-2667(19)30128-8

[6] Schubert I Ihle P Sabatowski R. Increase in opioid prescription in Germany between 2000 and 2009: a study-based on insurance data. Dtsch Arztebl Int. 2013;110(4):45–51. 23413387 10.3238/arztebl.2013.0045 PMC3570953

[7] Marschall U L’hoest H Radbruch L Häuser W. Long-term opioid therapy for chronic non-cancer pain in Germany. Eur J Pain. 2016;20(5):767–76. 10.1002/ejp.802 26492066

[8] Glaeske G Schicktanz C. BARMER GEK Arzneimittelreport 2012 [BARMER GEK Drug report 2012]. Vol 14. Asgard Verlagsservice GmbH; 2012.

[9] Alenezi A Yahyouche A Paudyal V. Current status of opioid epidemic in the United Kingdom and strategies for treatment optimisation in chronic pain. Int J Clin Pharm. 2021;43(2):318–22. 10.1007/s11096-020-01205-y PMC8079300 33252724

[10] International Narcotics Control Board. Report of the International Narcotics Control Board for 2017 [cited August 14, 2021]. Available from: http://www.incb.org/incb/en/publications/annual-reports/annual-report-2017.html

[11] US Department of Health and Human Services. What is the US opioid epidemic? [cited August 14, 2021]. Available from: https://www.hhs.gov/opioids/about-the-epidemic/index.html

[12] Haeuser W Ziegler D Viniol A, et al. Langzeitanwendung von Opioiden bei chronischen nicht-tumorbedingten Schmerzen (LONTS)–Leitlinie [Long-term use of opioids for chronic noncancer pain—LONTS guideline]. Berlin, Germany; 2020.

[13] McGettigan P Sly K O’Connell D Hill S Henry D. The effects of information framing on the practices of physicians. J Gen Intern Med. 1999;14(10):633–42. 10.1046/j.1525-1497.1999.09038.x PMC1496755 10571710

[14] Moxey A O’Connell D McGettigan P Henry D. Describing treatment effects to patients: how they are expressed makes a difference. J Gen Intern Med. 2003;18(11):948–59. 10.1046/j.1525-1497.2003.20928.x PMC1494946 14687282

[15] Jain BP. Number needed to treat and relative risk reduction. Ann Intern Med. 1998;128(1):72–3. 10.7326/0003-4819-128-1-199801010-00019 9424990

[16] Sethuraman R Cole C Jain D. Analyzing the effect of information format and task on cutoff search strategies. J Consum Psychol. 1994;3(2):103–36.

[17] Covey J. A meta-analysis of the effects of presenting treatment benefits in different formats. Med Decis Making. 2007;27(5):638–54. 10.1177/0272989X07306783 17873250

[18] Eddy DM. Probabilistic reasoning in clinical medicine: problems and opportunities. In: Kahneman D Slovic P Tversky A, eds. Judgment Under Uncertainty: Heuristics and Biases. Cambridge University Press; 1982:249–67.

[19] Casscells W Schoenberger A Graboys T. Interpretation by physicians of clinical laboratory results. N Engl J Med. 1978;299(18):999–1001. 692627 10.1056/NEJM197811022991808

[20] Bramwell R West H Salmon P. Health professionals’ and service users’ interpretation of screening test results: experimental study. BMJ. 2006;333(7562):284–86. 10.1136/bmj.38884.663102.AE PMC1526944 16840441

[21] Hoffrage U Gigerenzer G. Using natural frequencies to improve diagnostic inferences. Acad Med. 1998;73(5):538–40. 10.1097/00001888-199805000-00024 9609869

[22] Wegwarth O Gaissmaier W Gigerenzer G. Deceiving numbers: survival rates and their impact on doctors’ risk communication. Med Decis Making. 2011;31(3):386–94. 10.1177/0272989X10391469 21191123

[23] Wegwarth O Schwartz LM Woloshin S Gaissmaier W Gigerenzer G. Do physicians understand cancer screening statistics? A national survey of primary care physicians in the US. Ann Intern Med. 2012;156(5):340–9. 10.7326/0003-4819-156-5-201203060-00005 22393129

[24] Wegwarth O Gigerenzer G. US gynecologists’ estimates and beliefs regarding ovarian cancer screening’s effectiveness 5 years after release of the PLCO evidence. Sci Rep. 2018;8(1):17181. 30464251 10.1038/s41598-018-35585-z PMC6249225

[25] Wegwarth O Pashayan N. When evidence says no: gynecologists’ reasons for (not) recommending ineffective ovarian cancer screening. BMJ Qual Saf. 2020;29(6):521–4. 10.1136/bmjqs-2019-009854 PMC7323737 31704891

[26] Hoffmann TC Del Mar C. Clinicians’ expectations of the benefits and harms of treatments, screening, and tests. JAMA Intern Med. 2017;117(3):407–19. 10.1001/jamainternmed.2016.8254 28097303

[27] Gigerenzer G Gaissmaier W Kurz-Milcke E Schwartz LM Woloshin S. Helping doctors and patients make sense of health statistics. Psychol Sci Public Interest. 2007;8(2):53–96. 26161749 10.1111/j.1539-6053.2008.00033.x

[28] McDowell M Gigerenzer G Wegwarth O Rebitschek FG. Effect of tabular and icon fact box formats on comprehension of benefits and harms of prostate cancer screening: a randomized trial. Med Decis Making. 2019;39(1):41–56. 30799691 10.1177/0272989X18818166

[29] Schwartz LM Woloshin S Welch HG. Using a drug facts box to communicate drug benefits and harms. Ann Intern Med. 2009;150(8):516–27. 10.7326/0003-4819-150-8-200904210-00106 19221371

[30] Schwartz LM Woloshin S Welch HG. The drug facts box: providing consumers with simple tabular data on drug benefit and harm. Med Decis Making. 2007;27:655–62. 10.1177/0272989X07306786 17873258

[31] Denrell J. Adaptive learning and risk taking. Psychol Rev. 2007;114(1):177–87. 10.1037/0033-295X.114.1.177 17227186

[32] Hertwig R. Die Bedeutung von beschreibungsbasiertem versus erfahrungsbasiertem Risikoverhalten für die Arzneimitteltherapiesicherheit. In: Grandt D Lappe V Schubert I, eds. Arzneimittelreport 2018. Vol 10. BARMER; 2018:154–9.

[33] Newell BR Rakow T Yechiam E Sambur M. Rare disaster information can increase risk-taking. Nat Clim Change. 2015;6:158–61.

[34] Wulff DU Mergenthaler-Canseco M Hertwig R. A meta-analytic review of two modes of learning and the description-experience gap. Psychol Bull. 2018;144(2):140–76. 10.1037/bul0000115 29239630

[35] Hertwig R Erev I. The description experience gap in risky choice. Trends Cogn Sci. 2009;13(12):517–23. 10.1016/j.tics.2009.09.004 19836292

[36] Barron G Leider S Stack J. The effect of safe experience on a warnings’ impact: sex, drugs, and rock-n-roll. Organ Behav Hum Decis Process. 2008;106(2):125–42.

[37] Kaufmann C Weber M Haisley E. The role of experience sampling and graphical displays on one’s investment risk appetite. Manage Sci. 2013;59(2):323–40.

[38] Armstrong B Spaniol J. Experienced probabilities increase understanding of diagnostic test results in younger and older adults. Med Decis Making. 2017;37(6):670–9. 10.1177/0272989X17691954 28199179

[39] Hogarth RM Soyer E. Sequentially simulated outcomes: kind experience versus nontransparent description. J Exp Psychol Gen. 2011;140(3):434–63. 10.1037/a0023265 21639669

[40] Wegwarth O Spies C Schulte E, et al. Experiencing the risk of overutilising opioids among patients with chronic non-cancer pain in ambulatory care (ERONA): the protocol of an exploratory, randomised controlled trial. BMJ Open. 2020;10(9):e037642. 10.1136/bmjopen-2020-037642 PMC7476567 32895283

[41] Cochrane. Methods Rapid Reviews [cited August 14, 2021]. Available from: https://methods.cochrane.org/rapidreviews/

[42] Nury E Schmucker C Nagavci BL, et al. Efficacy and safety of long-term opioid therapy in patients with chronic non-cancer pain: a systematic review and meta-analysis. Pain. Published online July 28, 2021. doi:10.1097/j.pain.0000000000002423

[43] Johnson EJ Payne JW Schkade DA Bettman JR. Monitoring Information Processing and Decisions: The Mouselab System. Center for Decision Studies, Fuqua School of Business, Duke University (Unpublished manuscript); 1989.

[44] Caverly TJ Prochazka AV Combs BP, et al. Doctors and numbers: an assessment of the Critical Risk Interpretation Test. Med Decis Making. 2015;35(4):512–24. 10.1177/0272989X14558423 25378297

[45] Gollwitzer PM Sheeran P. Implementation intentions and goal achievement: a meta-analysis of effects and processes. Adv Exp Soc Psychol. 2006;38:69–119.

[46] Stang A. Nonresponse research: an underdeveloped field in epidemiology. Eur J Epidemiol. 2003;18:929–31. 10.1023/a:1025877501423 14598921

[47] Hoffmann W Terschüren C Holle R, et al. The problem of response in epidemiologic studies in Germany (Part II) [in German]. Gesundheitswesen. 2004;66(8/9):482–91. 10.1055/s-2004-813094 15372348

[48] Latza U Stang A Bergmann M, et al. The problem of response in epidemiologic studies in Germany (Part I) [in German]. Gesundheitswesen. 2004;67(5):326–36. 10.1055/s-2004-813093 15141353

