# Supplementary material for: Educating Pharmacists on the Risks of Strong Opioids With Descriptive and Simulated Experience Risk Formats: A Randomized Controlled Trial
Source: MDM Policy Pract. 2021 Sep 27;6(2):23814683211042832. doi: 10.1177/23814683211042832 (PMC8482350; doi:10.1177/23814683211042832)
Supplement: sj-docx-1-mpp-10.1177_23814683211042832 – Supplemental material for Educating Pharmacists on the Risks of Strong Opioids With Descriptive and Simulated Experience Risk Formats: A Randomized Controlled Trial [file sj-docx-1-mpp-10.1177_23814683211042832.docx]

**Electronic Supplementary Material**

Wegwarth, O., Wind, S., Goebel, E., Spies, C., Meerpohl, J. J., Schmucker, C., Schulte, E., Neugebauer, E., & Hertwig, R. **Educating Pharmacists on the Risks of Strong Opioids with Descriptive And Simulated Experience Risk Formats: a randomized-controlled trial.**

***Content***

1. *Results.*
   1. Regression table 1 reporting the association between improvement in objective risk perception and the factors medical risk literacy, gender, years in practice, and region of practice
   2. Regression table 2 reporting the association between improvement in subjective risk perception and the factors medical risk literacy, gender, years in practice, and region of practice
   3. Regression table 3 reporting the association between intended change in counselling behaviour and the factors medical risk literacy, gender, years in practice, and region of practice
   4. Regression tables 4.1 to 4.4 reporting the association between consistency in intended and actual counselling behaviour and the factors medical risk literacy, gender, years in practice, region of practice, and intervention by alternative treatment option
   5. CONSORT Flow chart

**Regression table 1 |** Association between improvement in objective risk perception and the factors medical risk literacy and demographic variables

| **Variables** | **Improvement in objective risk perception**  *Reference class: 2 or more risk estimates correct after intervention* | | |
| --- | --- | --- | --- |
|  | Odds ratio | 95% CI | *p* |
| **Medical Risk Literacy*** |  |  |  |
| ***Low literacy*** (0–2 of 5 questions correct) (reference class: moderate/excellent literacy [3–5 correct]) | 0.99 | 0.47–2.08 | .98 |
| **Gender** |  |  |  |
| ***Female*** (reference class: male) | 1.53 | 0.90–2.60 | .12 |
| **Years in Practice** |  |  |  |
| ***< 10 years*** | 0.41 | 0.16–1.02 | .06 |
| ***10–19 years*** | 0.71 | 0.33–1.49 | .36 |
| ***20–29 years*** | 0.97 | 0.46–2.04 | .94 |
| Reference class: ≥ 30 years |  |  |  |
| ***Region of Practice*** |  |  |  |
| ***North Germany*** | 1.08 | 0.54–2.18 | .83 |
| ***South Germany*** | 1.20 | 0.60–2.41 | .61 |
| ***East Germany*** | 1.02 | 0.50–2.09 | .95 |
| Reference class: West **Germany** |  |  |  |

* Based on adapted version of: Caverly TJ et al. Doctors and numbers: an assessment of the Critical Risk Interpretation Test. *Med Decis Making.* 2015;*35:*512–524.

**Regression table 2 |** Association between improved subjective risk perception and the factors medical risk literacy and demographic variables

| **Variables** | **Improvement in subjective risk perception**  *Reference class: Change towards a category suggesting the perception of harms (versus no change)* | | |
| --- | --- | --- | --- |
|  | Odds ratio | 95% CI | *p* |
| **Medical Risk Literacy*** |  |  |  |
| ***Low literacy*** (0–2 of 5 questions correct) (reference class: moderate/excellent literacy [3–5 correct]) | 0.43 | 0.20–0.91 | .28 |
| **Gender** |  |  |  |
| ***Female*** (reference class: male) | 0.84 | 0.46–1.54 | .58 |
| **Years in Practice** |  |  |  |
| ***< 10 years*** | 0.74 | 0.26–2.16 | .59 |
| ***10–19 years*** | 0.90 | 0.38–2.15 | .81 |
| ***20–29 years*** | 0.94 | 0.40–2.22 | .88 |
| Reference class: ≥ 30 years |  |  |  |
| ***Region of Practice*** |  |  |  |
| ***North Germany*** | 0.67 | 0.29–1.56 | .35 |
| ***South Germany*** | 0.56 | 0.25–1.27 | .17 |
| ***East Germany*** | 0.98 | 0.40–2.42 | .95 |
| Reference class: West **Germany** |  |  |  |

* Based on adapted version of: Caverly TJ et al. Doctors and numbers: an assessment of the Critical Risk Interpretation Test. *Med Decis Making.* 2015;*35:*512–524.

**Regression table 3 |** Association between intention to actively counsel patients with chronic noncancer pain on treatment alternatives and the factors medical risk literacy and demographic variables

| **Variables** | **Intention to actively counsel patients on treatment alternatives**  *Reference class: yes* | | |
| --- | --- | --- | --- |
|  | Odds ratio | 95% CI | *p* |
| **Medical Risk Literacy*** |  |  |  |
| ***Low literacy*** (0–2 of 5 questions correct) (reference class: moderate/excellent literacy [3–5 correct]) | 0.84 | 0.41–1.71 | .63 |
| **Gender** |  |  |  |
| ***Female*** (reference class: male) | 1.06 | 0.65–1.74 | .81 |
| **Years in Practice** |  |  |  |
| ***< 10 years*** | 1.12 | 0.47–2.69 | .80 |
| ***10–19 years*** | 1.41 | 0.70–2.84 | .34 |
| ***20–29 years*** | 1.03 | 0.51–2.06 | .94 |
| Reference class: ≥ 30 years |  |  |  |
| ***Region of Practice*** |  |  |  |
| ***North Germany*** | 0.78 | 0.40–1.53 | .46 |
| ***South Germany*** | 1.12 | 0.59–2.16 | .73 |
| ***East Germany*** | 0.93 | 0.47–1.83 | .82 |
| Reference class: West **Germany** |  |  |  |

* Based on adapted version of: Caverly TJ et al. Doctors and numbers: an assessment of the Critical Risk Interpretation Test. *Med Decis Making.* 2015;*35:*512–524.

**Regression tables 4.1 to 4.4 |** Consistency between intended and actual counselling behaviour by treatment option. (Consistency was assumed if there was no decrease between the intended and the actual proportion of patients recommended the respective therapy.)

**Regression table 4.1 |** Association between consistency in intended and actual counselling of patients with chronic noncancer pain on physiotherapy and the factors medical risk literacy and demographic variables

| **Variables** | **Consistency between intended and actual counselling on physiotherapy**  *Reference class: yes* | | |
| --- | --- | --- | --- |
|  | Odds ratio | 95% CI | *p* |
| **Medical Risk Literacy*** | 0.24 | 0.03–1. 81 | 0.17 |
| **Gender** | 1.53 | 0.65–3.62 | 0.33 |
| **Age** | 1.04 | 0.54–2.00 | 0.91 |
| **Region of Practice** | 1.13 | 0.78–1.62 | 0.53 |
| **Intervention** | 2.92 | 1.18–7.21 | 0.02 |

* Based on adapted version of: Caverly TJ et al. Doctors and numbers: an assessment of the Critical Risk Interpretation Test. *Med Decis Making.* 2015;*35:*512–524.

**Regression table 4.2 |** Association between consistency in intended and actual counselling of patients with chronic noncancer pain on lifestyle changes and the factors medical risk literacy and demographic variables

| **Variables** | **Consistency between intended and actual counselling on lifestyle changes**  *Reference class: yes* | | |
| --- | --- | --- | --- |
|  | Odds ratio | 95% CI | *p* |
| **Medical Risk Literacy*** | 0.50 | 0.06–3.94 | 0.51 |
| **Gender** | 0.88 | 0.28–2.77 | 0.83 |
| **Age** | 1.29 | 0.53–3.10 | 0.58 |
| **Region of Practice** | 0.93 | 0.58–1.50 | 0.77 |
| **Intervention** | 1.01 | 0.34–2.95 | 0.99 |

* Based on adapted version of: Caverly TJ et al. Doctors and numbers: an assessment of the Critical Risk Interpretation Test. *Med Decis Making.* 2015;*35:*512–524.

**Regression table 4.3 |** Association between consistency in intended and actual counselling of patients with chronic noncancer pain on psychotherapy and the factors medical risk literacy and demographic variables

| **Variables** | **Consistency between intended and actual counselling on psychotherapy**  *Reference class: yes* | | |
| --- | --- | --- | --- |
|  | Odds ratio | 95% CI | *p* |
| **Medical Risk Literacy*** | 1.26 | 0.40–3.94 | 0.70 |
| **Gender** | 1.28 | 0.53–3.06 | 0.58 |
| **Age** | 1.49 | 0.77–2.88 | 0.23 |
| **Region of Practice** | 1.03 | 0.71–1.48 | 0.88 |
| **Intervention** | 2.42 | 1.01–5.79 | 0.04 |

* Based on adapted version of: Caverly TJ et al. Doctors and numbers: an assessment of the Critical Risk Interpretation Test. *Med Decis Making.* 2015;*35:*512–524.

**Regression table 4.4 |** Association between consistency in intended and actual counselling of patients with chronic noncancer pain on lifestyle changes and the factors medical risk literacy and demographic variables

| **Variables** | **Consistency between intended and actual counselling on multimodal therapy**  *Reference class: yes* | | |
| --- | --- | --- | --- |
|  | Odds ratio | 95% CI | *p* |
| **Medical Risk Literacy*** | 1.26 | 0.25–6.43 | 0.78 |
| **Gender** | 1.54 | 0.51–4.62 | 0.44 |
| **Age** | 1.03 | 0.48–2.23 | 0.93 |
| **Region of Practice** | 0.80 | 0.51–1.25 | 0.33 |
| **Intervention** | 1.46 | 0.52–4.13 | 0.47 |

* Based on adapted version of: Caverly TJ et al. Doctors and numbers: an assessment of the Critical Risk Interpretation Test. *Med Decis Making.* 2015; *35:*512–524.


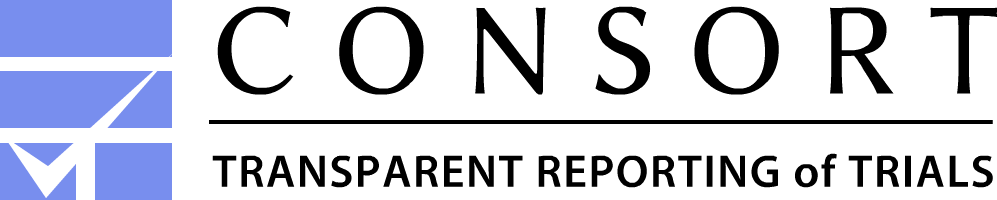


**CONSORT 2010 Flow Diagram**

Lost to follow-up (n= 89)

♦ Excluded from analysis because of non-
 eligibility for T2 (n= 57)

♦ Did not response to follow-up invitation
 (n = 32)

Lost to follow-up (n= 78)

♦ Excluded from analysis because of non-
 eligibility for T2 (n= 59 )

♦ Did not response to follow-up invitation
 (n = 19)

Analyzed (n= 61)

Analyzed (n = 72)

Analysis T2

Follow-up (T2)

Analyzed for wave 1 (T1) (n= 150)

Analyzed for wave 1 (T1) (n= 150)

Assessed for eligibility

(n= 2,679)

Analysis T1

Enrollment

Allocated to descriptive intervention (n= 150)

♦ Received allocated intervention (n= 150)

♦ Did not receive allocated intervention (n= 0 )

Allocation

Allocated to simulated experience intervention (n= 150 )

♦ Received allocated intervention (n= 150 )

♦ Did not receive allocated intervention (n= 0)

Randomized (n= 300)

Excluded (n= 2,379)

♦  Not meeting inclusion criteria (n= 23)

♦  Did not finish the survey (n= 69)

♦  Did not respond (n= 2,287)
